# Estrogen receptor-α and progesterone receptor are expressed in label-retaining mammary epithelial cells that divide asymmetrically and retain their template DNA strands

**DOI:** 10.1186/bcr1538

**Published:** 2006-08-01

**Authors:** Brian W Booth, Gilbert H Smith

**Affiliations:** 1 Mammary Biology and Tumorigenesis Laboratory, National Cancer Institute, National Institute of Health, Bethesda, Maryland, USA

## Abstract

**Introduction:**

Stem cells of somatic tissues are hypothesized to protect themselves from mutation and cancer risk through a process of selective segregation of their template DNA strands during asymmetric division. Mouse mammary epithelium contains label-retaining epithelial cells that divide asymmetrically and retain their template DNA.

**Method:**

Immunohistochemistry was used in murine mammary glands that had been labeled with [^3^H]thymidine during allometric growth to investigate the co-expression of DNA label retention and estrogen receptor (ER)-α or progesterone receptor (PR). Using the same methods, we investigated the co-localization of [^3^H]thymidine and ER-α or PR in mammary tissue from mice that had received treatment with estrogen, progesterone, and prolactin subsequent to a long chase period to identify label-retaining cells.

**Results:**

Label-retaining epithelial cells (LRECs) comprised approximately 2.0% of the entire mammary epithelium. ER-α-positive and PR-positive cells represented about 30–40% of the LREC subpopulation. Administration of estrogen, progesterone, and prolactin altered the percentage of LRECs expressing ER-α.

**Conclusion:**

The results presented here support the premise that there is a subpopulation of LRECs in the murine mammary gland that is positive for ER-α and/or PR. This suggests that certain mammary LRECs (potentially stem cells) remain stably positive for these receptors, raising the possibility that LRECs comprise a hierarchy of asymmetrically cycling mammary stem/progenitor cells that are distinguished by the presence or absence of nuclear steroid receptor expression.

## Introduction

Dividing adult somatic stem cells are postulated to protect themselves from mutation and cancer risk by segregation of their template DNA strands through a process known as asymmetric division [1-3]. This property is deemed to protect long-lived stem cells from errors incurred during DNA replication that lead to mutagenesis [2]. In the mouse mammary gland, label-retaining epithelial cells (LRECs) in the ducts divide asymmetrically and retain their template DNA strands [4]. In addition, more than 80% of these LRECs remain in the cell cycle, dividing actively as evidenced by their incorporation of a second DNA label after a 48-hour pulse. Ductal morphogenesis occurs between the third and tenth weeks of age in the mouse, during which time the mammary gland is rapidly proliferating and differentiating. Mammary epithelial stem cells contribute to this development of the mammary gland through both asymmetric and symmetric division: asymmetric division for the generation of transit-amplifying and progenitor cells that subsequently differentiate; and symmetric division for self-renewal and stem cell expansion. In the mature mouse the mammary epithelium exists in a state of relative proliferative quiescence before pregnancy. Tissue homeostasis is maintained by stem cells positioned throughout the mammary ductal system.

The importance of estrogen-mediated and progesterone-mediated responses for normal mammary growth and development and during mammary carcinogenesis is well recognized [5]. Here, we examine LRECs in mouse mammary epithelium for expression of estrogen receptor (ER)-α and PR. We found that epithelial cells with these characteristics are labeled during mammary ductal development, and that after a long chase period they continue to retain both the original DNA label and expression of PR and ER-α. This observation suggests that a proportion of asymmetrically cycling LRECs is permanently steroid hormone receptor positive and represents a unique subset of stem/progenitor cells among the mouse mammary epithelium.

## Materials and methods

### Experimental plan

The experimental plan was reported previously [4]. Briefly, the experiment was begun when the FVB/N mice were exactly 32 days of age. Sixteen mice were used in each experiment. The mice received daily intraperitoneal injections of 1.0 μg estradiol, followed 2 hours later by an intraperitoneal injection of [^3^H]thymidine of either 25 or 50 μCu for 5 consecutive days. Two animals were removed for tissue analysis following the final [^3^H]thymidine injection to determine the number of mammary cells that were labeled. The number 3, 4, 8, and 9 mammary glands were collected. The small intestine from each animal was excised and bundled to provide a positively labeled control for autoradiography as an indicator of successful incorporation of the nuclear label. Subsequently, estradiol (1.0 μg) was given every other day for 3 weeks to promote mammary growth. Upon cessation of estradiol treatment (the ninth week of life), the animals were held for 2 weeks; at the end of the 11th week of life, tissues were removed from two animals to determine the number and location of long-label-retaining mammary cells.

The remaining mice were placed in three groups and treated as follows (daily doses given): group I received 1.0 μg estradiol; group II received 1.0 μg estradiol and 1.0 mg progesterone; and group III received estradiol and progesterone (same doses as groups I and II) plus 0.5 μg/g body weight prolactin.

Each group received 1.0 mg of 5-bromo-deoxyuridine (5BrdU) intraperitoneally, on day 1 and day 2 of hormone treatment. All hormone treatments were maintained for 5 consecutive days. One animal from each group was removed for tissue analysis after the third day of hormone treatment. The remaining animals were analyzed 3 days following the final hormone treatment (6 days after the final 5BrdU injection).

The protocols and procedures used in the experiments were reviewed and approved by the Animal Care and Use Committee at the NCI-Frederick. Housing and care during the experimental period conformed to the guidlines provided by the US National Institutes of Health.

### Autoradiography and immunochemistry

All immunohistochemistry was performed after autoradiographic exposure, the sections were deparaffinized and rehydrated, and endogenous peroxidase was inactivated with 1% hydogen peroxide in methanol for 30 min. Antibodies used were anti-progesterone receptor 1:75 (clone A009B; Dako USA, Carpinteria, CA, USA) and anti-ER-α 1:50 (clone MC-20; Santa Cruz Biotech, Santa Cruz, CA, USA). Antigen retrieval was accomplished according to the direction of the manufacturer. Negative tissue controls were included in all immunohistochemical analyses. Sections were counter-stained with hematoxylin or nuclear fast red after immunostaining.

For autoradiography, 5–6 μm sections were cut, placed upon slides, de-waxed, rehydrated through ethanol, and subsequently dipped in Kodak (Rochester, New York, USA) NTB-2 liquid emulsion diluted 1:1 with distilled water. After drying, the slides were stored in lightproof slide boxes at constant humidity and temperature for 20 days. After exposure, the slides were developed in Kodak D-19, washed in distilled water, and fixed in Kodak rapid fixer diluted 1:1 with distilled water. After staining and mounting, the slides were observed and evaluated for autoradiographic grains and for immunostaining. Images were recorded with Kodak digital microscopy documentation system 290 and edited with Adobe Photoshop 7.0.

Determination of autoradiographic grain counts in epithelial cells was made by counting the grains over at least 100 LRECs in sections from each of the four mammary glands taken from each experimental mouse. At least 500 labeled cells were counted in each of these sections. At least 3000 nuclei were examined in each slide. Examination of autoradiographic slides from these tissues that were stained for PR and ER revealed similar numbers of autoradiographic grains over LREC nuclei.

### Statistical analysis

The SigamStat software package (Systat Software, Chicago, Illinois, USA) was used for all analyses. Data were considered significant at *P *< 0.05. Representative data are presented as mean ± standard error of the mean.

## Results

**Figure 1:**
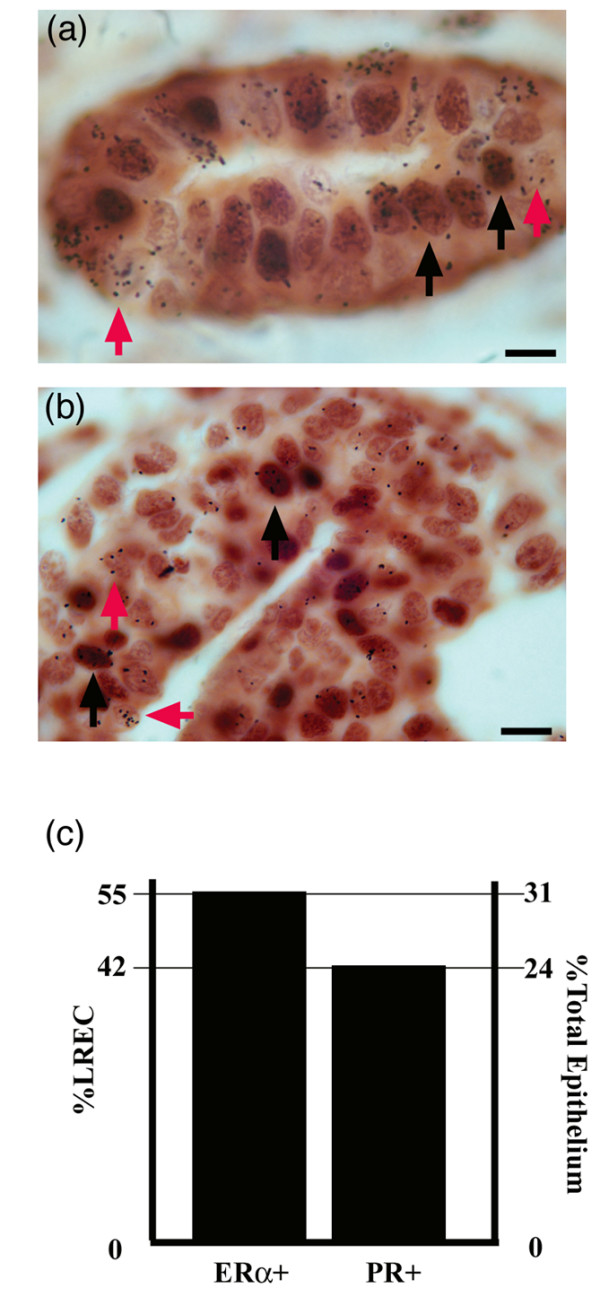
ER-α-positive and PR-positive epithelial cells are in cycle in during allometric growth. **(a) **ER-α and [^3^H]thymidine are detected in mammary tissue. ER-α-positive staining is indicated with black arrowheads and [^3^H]thymidine incorporation is indicated with red arrowheads. **(b) **PR and [^3^H]thymidine are detected in mammary tissue. PR-positive staining is indicated with black arrowheads and [^3^H]thymidine incorporation is indicated with red arrowheads. **(c) **Graphical depiction of the number of cells that were double positive for [^3^H]thymidine incorporation and ER-α or PR (left scale) and the percentage of cells that were ER-α or PR positive within epithelium. Scale bars = 5 μm. ER, estrogen receptor; PR, progesterone receptor.

**Figure 2:**
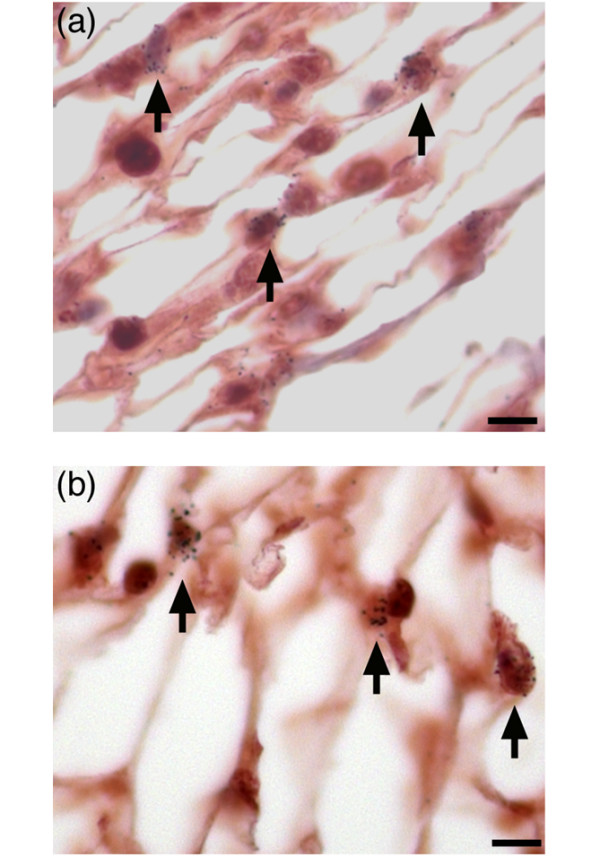
Stromal cells proliferate in pubertal mammary tissue. The nuclear label [^3^H]thymidine is detected in mammary tissue that has been processed for **(a) **ER-α or **(b) **PR. Black arrows designate stromal cells that are [^3^H]thymidine positive, indicating cells that have recently undergone cell cycle. Scale bars = 5 μm. ER, estrogen receptor; PR, progesterone receptor.

To determine whether ER-α-positive and PR-positive mammary epithelial cells were in cycle during allometric growth, 5-week-old mice were injected with [^3^H]thymidine during the 5th week of life for 5 days. After a 2-day pause a group was sacrificed to determine the efficiency of labeling. The mammary glands were processed for either ER-α or PR immunostaining and [^3^H]thymidine. Figure 1 demonstrates that 55% of the epithelial cells were in cycle, as determined by the presence of [^3^H]thymidine label, and that within this group of cycling cells 31% were ER-α positive (Figure 1a) and 24% were PR positive (Figure 1b). The labeled ER-α-positive and PR-positive cells were represented equally among the body cells of terminal end buds and within the epithelium of the subtending ducts. It is noteworthy that numerous ER-α-negative and PR-negative cells were also labeled during the 5-day labeling period. Numerous cells within the surrounding stroma were also positive for [^3^H]thymidine incorporation (Figure 2), indicating that stromal cells as well as epithelial cells were proliferating in the pubertal gland.

**Figure 3:**
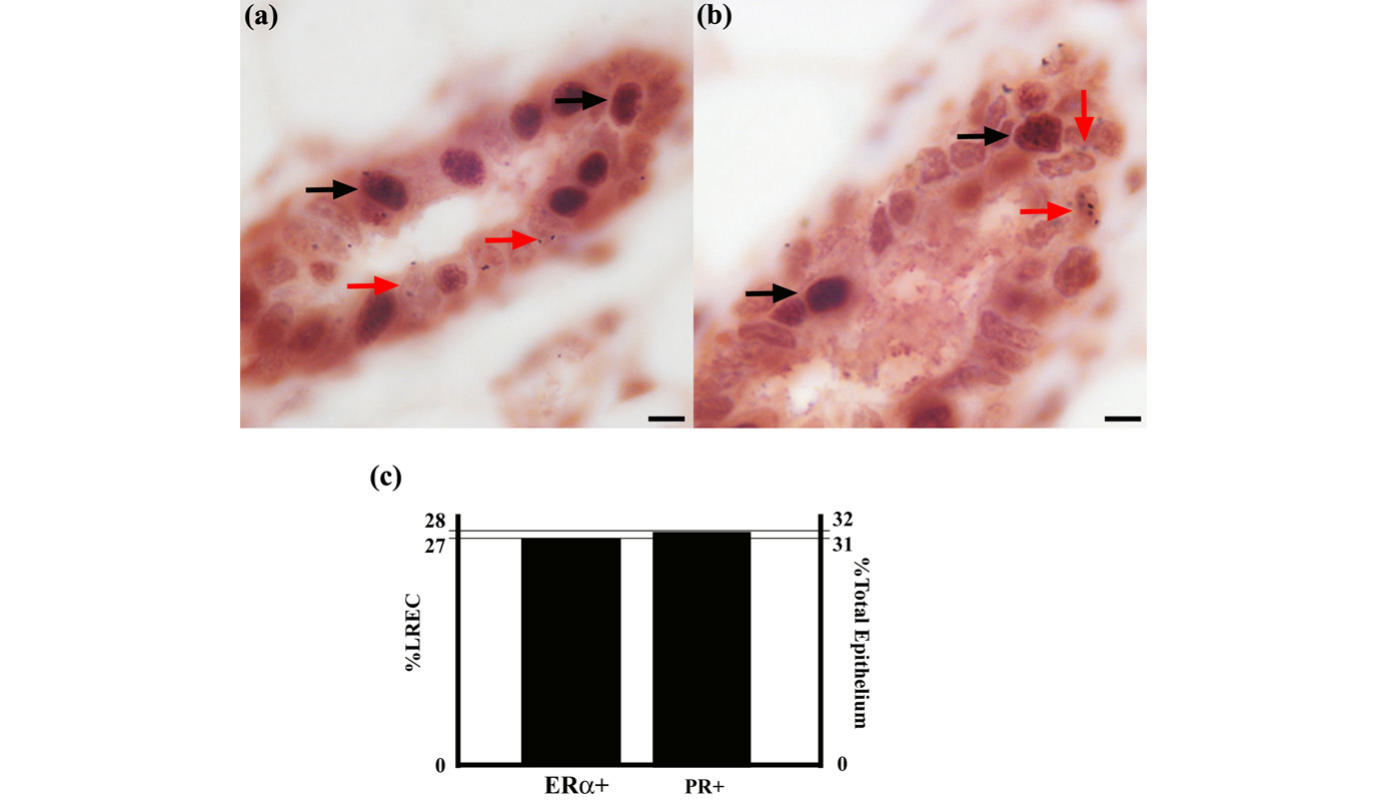
A subpopulation of LRECs is ER-α positive or PR positive. **(a) **ER-α and [^3^H]thymidine are detected in mammary tissue. LRECs that are double positive for ER-α and [^3^H]thymidine incorporation are indicated with black arrows. Cells that are LRECs alone are indicated with red arrowheads. **(b) **PR and [^3^H]thymidine are detected in mammary tissue. LRECs that are double positive for PR and [^3^H]thymidine incorporation are indicated with black arrows. Cells that are LRECs alone are indicated with red arrows. **(c) **Graphical depiction of the number of cells that are double positive for [^3^H]thymidine incorporation and ER-α or PR (left scale) and percentage of cells that are ER-α or PR positive within epithelium. Scale bars = 5 μm. ER, estrogen receptor; LREC, label-retaining epithelial cell; PR, progesterone receptor.

**Figure 4:**
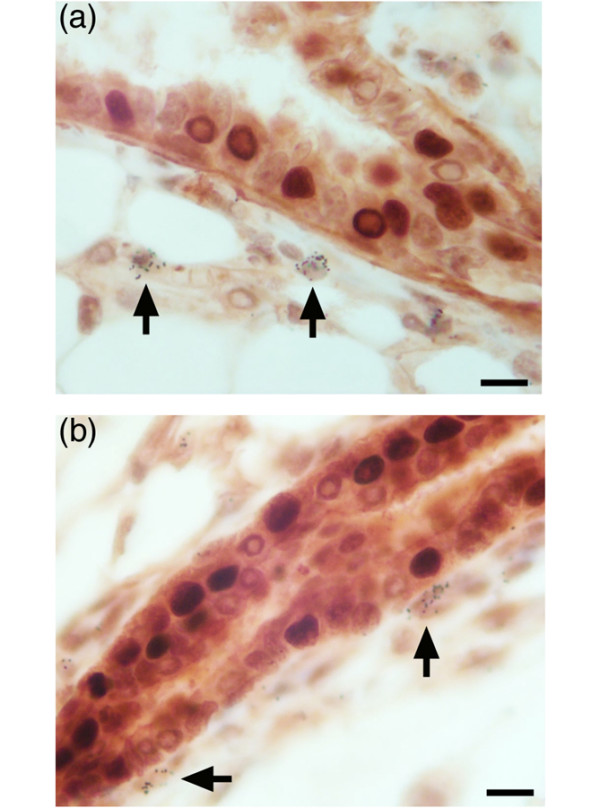
Periductal stromal cells retain nuclear label. The nuclear label [^3^H]thymidine is detected in mammary tissue that has been processed for **(a) **ER-α or **(b) **PR following a 7-week chase period. Black arrows indicate periductal stromal cells that are [^3^H]thymidine positive. Scale bars = 5 μm. ER, estrogen receptor; PR, progesterone receptor.

A majority of mammary epithelial cells were labeled with [^3^H]thymidine during the 5 days of allometric growth, as demonstrated by the number of labeled cells. To determine whether any of the [^3^H]thymidine labeled cells retained label as well as ER-α or PR, we allowed a 6-week chase period after the initial [^3^H]thymidine injections, during which duct morphogenesis was completed. Seven weeks after the final [^3^H]thymidine injection, animals were sacrificed and the mammary glands processed for ER-α or PR and [^3^H]thymidine label (Figure 3). LRECs were detected in both luminal and basal positions (about 2% of epithelial cells were LRECs). Through cell counts we determined that 27% (about 5.4/1000 total epithelial cells) of LRECs (about 21/1000) were ER-α positive and 28% (about 5.6/1000 total epithelial cells) were PR positive. In the total epithelial population 31% (about 310/1000) were ER-α positive whereas 32% (about 320/1000) were PR positive (Table 1). Of interest is the similarity of the relative percentage of labeled cells that expressed ER-α or PR after the prolonged chase compared with the percentage of ER-α-positive and PR-positive cells found labeled after the original 5-day labeling period. This suggests that steroid receptor positive and negative LRECs may be developed simultaneously during allometric mammary growth. Label-retaining periductal stromal cells were also present in sections from the chased tissue (Figure 4).

In order to determine the number of LRECs that were in cycle, 5BrdU was given for 2 days. Under these conditions, most of the LRECs (about 80%) were doubly positive for [^3^H]thymidine and 5BrdU, indicating that they were actively in cycle [4]. On the other hand, none of the label-retaining stromal cells were doubly positive, indicating that they were out of cycle.

**Figure 5:**
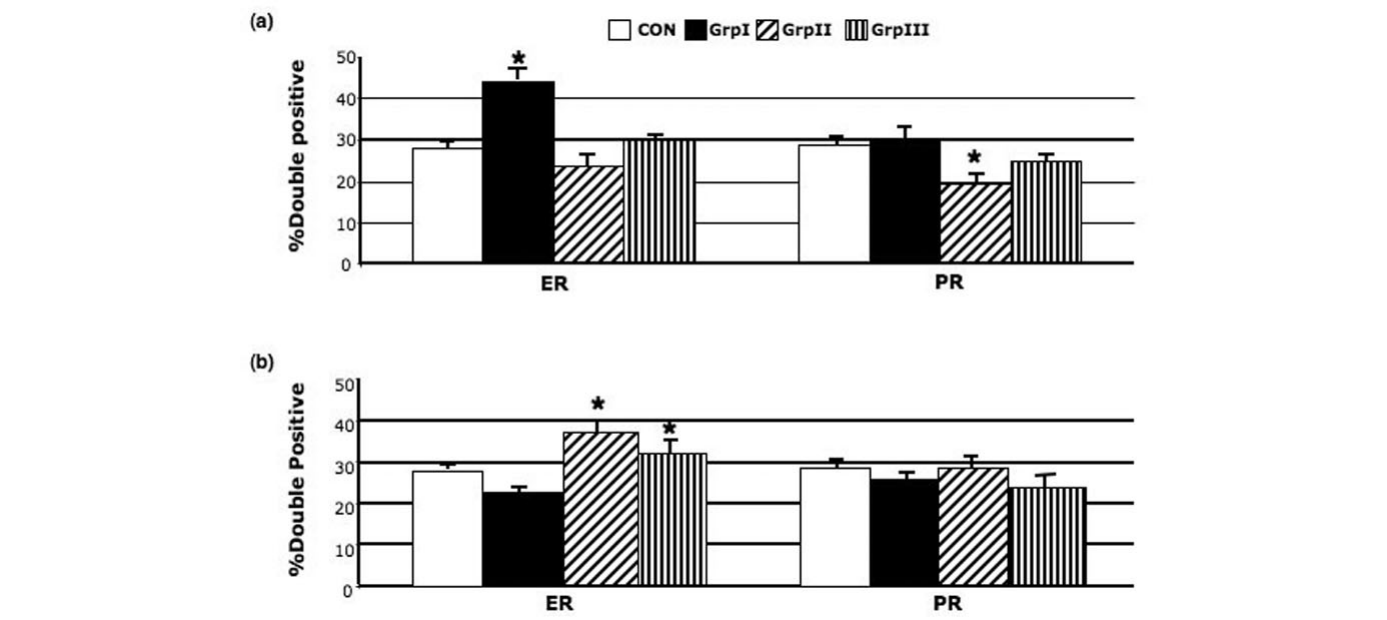
Graphical depiction of percentage of LRECs that were either ER-α positive or PR positive after hormone treatments. All animals received [^3^H]thymidine at 5 weeks of age. Control animals (Con; white bar) received only [^3^H]thymidine. After a 7-week chase group I (GrpI; black bar) received estradiol injections; group II (GrpII; diagonal hatches) animals received estradiol and progesterone; and group III (GrpIII; vertical hatches) received estradiol, progesterone, and prolactin. **(a) **Results after 3-day hormone treatment and **(b) **results obtained after 5-day hormone treatment and 2-day recovery period. Error bars indicate the standard error of the mean. *P < 0.05, versus Con. ER, estrogen receptor; LREC, label-retaining epithelial cell; PR, progesterone receptor.

During this treatment mice were divided into three groups and given injections of estradiol (group I), estradiol plus progesterone (group II), or estradiol plus progesterone plus prolactin (group III). The ratio of LRECs to total epithelium remained unchanged in all groups. ER-α and PR staining was performed on tissue from the third and eighth day (hormones were discontinued on day 5). The results indicate that following a 2-day administration of estradiol, the percentage of LRECs that were also ER-α positive rose to approximately 44% (*P *< 0.05) whereas the percentage of PR-positive LRECs was unchanged (Figure 5a). The combination of estradiol plus progesterone for 2 days brought about no change in ER-α-positive LRECs but it resulted in a decrease in PR-positive LRECs to 19% (*P *< 0.05). No significant changes in ER-α or PR expression in LRECs were observed in any group III mice. Counts performed on tissue harvested 3 days after the final hormone treatment revealed that the combination of estradiol and progesterone had increased expression of ER-α in LRECs to 37% (*P *< 0.05) whereas estradiol plus progesterone plus prolactin had increased ER-α expression in LRECs to 32% (*P *< 0.05; Figure 5b). In mice that had received estradiol alone the numbers of ER-α-positive LRECs were not significantly different from those in untreated animals. No significant changes in the number of PR-positive LRECs were observed in any treatment group 3 days after the final hormone treatment. These data are summarized in Table 2. The percentage of ER-α-positive or PR-positive cells overall among the total mammary epithelium exhibited no significant alterations (Table 1).

## Discussion

This study demonstrates a subset of murine mammary epithelial cells that is long label retaining and expresses ER-α or the PR. Previously, it was shown that more than 80% of LRECs are actively traversing the cell cycle, despite retention of the original DNA label [4]. The retention of the original DNA label over many weeks serves to establish the validity of our conclusion that the LRECs represent long-lived cells. Whether these label-retaining cells remain ER-α and/or PR positive from their inception through the entire chase period is not possible to determine. Nevertheless, we observed a high percentage of ER-α and PR positive, [^3^H]thymidine labeled, epithelial cells following the original labeling period. In addition, we did not establish whether ER-α and PR are simultaneously expressed in these cells. Recent data indicate that the expression of active transforming growth factor (TGF)-β_1 _restrains ER-α-positive cells among the mouse mammary epithelium from entering the cell cycle [6]. In that report ER-α, PR, and activated TGF-β_1 _were shown to colocalize in individual mammary epithelial cells. This TGF-β_1_-mediated restraint appeared to be differentially regulated in pubertal mice as compared with mammary tissue in adult glands, with ER-α-positive cells in pubertal tissues showing less responsiveness to depletion of activated TGF-β_1 _than ER-negative cells. This is consistent with our observation of numerous ER-α-positive/[^3^H]thymidine-positive epithelial cells in the mammary tissue from 5-week-old females following a 5-day application of [^3^H]thymidine. In addition, ectopic expression of constitutively active TGF-β_1 _from the whey acidic protein promoter in mammary epithelium during allometric growth of the glands results in early growth senescence in mammary transplants from this tissue, suggesting a derangement in epithelial stem cell self-renewal.

These observations taken together suggest the possible existence of an ER-α-positive mammary epithelial stem/progenitor cell. In agreement with this hypothesis, the expression of constitutively active TGF-β_1 _in mammary alveolar progenitors during lobulogenesis inhibits their subsequent capacity to proliferate on transplantation into cleared mammary fat pads [7].

Clarke and coworkers [8] recently suggested in a human model that steroid receptor-positive cells are stem cells that self-renew through asymmetric division and produce transit amplifying and differentiated epithelial progeny. We demonstrated in a murine model that LRECs divide asymmetrically and are capable of re-populating new stem cell niches, indicating that LRECs possess stem cell qualities [4]. The association of steroid receptors and LRECs was reported previously [9]. In agreement with that report, we observed that approximately 50% of the epithelium is cycling during allometric growth, as determined by label uptake. Unlike the findings reported by Zeps and coworkers [9], we observed numerous ER-α-negative label-retaining cells as well as ER-α-positive LRECs. However, there were significant differences in the labeling protocols that were used in our respective studies, and this in all likelihood is the basis for the difference in our individual findings. Zeps and coworkers labeled 12-week-old mature females intensively by giving [^3^H]thymidine label three times an hour apart and then sought to detect ER-α-positive long-label-retaining cells after a 2-week chase. Under these conditions in mature females, no ER-α-negative label-retaining cells were observed following the chase. We labeled immature females in their fourth week of life during active ductal growth and elongation.

Similar to Zeps and coworkers, we found that both ER-α-positive and ER-α-negative cells were labeled in the developing glands. Both ER-α-negative and ER-α-positive long-label-retaining cells were present after 7–8 weeks in our study. We believe that this is because we labeled the dividing epithelial population continuously for 5 consecutive days during stem/progenitor cell expansion in the growing ductal epithelium, whereas Zeps and coworkers labeled intensively for 3 hours at estrus in mature 12-week-old females followed by a 2-week chase. The persistence of ER-α-positive label-retaining cells in this experiment after 2 weeks may be accounted for by the selective symmetric expansion of ER-α-positive LRECs at estrus in the mature mammary epithelium. A further suggestion is that ER-α-positive/PR-positive LRECs and ER-α-negative/PR-negative LRECs may represent different subsets of long-label-retaining stem/progenitors cells among the mammary epithelium.

The observation that estrogen upregulates ER-α expression without an accompanying increase in PR expression in LRECs is novel. The animals had not received [^3^H]thymidine for more than 7 weeks, precluding the formation of new LRECs by additional incorporation of label. Cellular proliferation (i.e. symmetric cellular division) of ER-α-positive LRECs is a possible explanation. It was recently reported that ER-positive cells in monkeys proliferate in response to estrogen [10]. The mechanism by which estrogen induces proliferation is through the release of growth mediators by ER-positive cells that act in a paracrine manner on ER-negative cells initiating the cell cycle [11]. This cannot be the explanation in this study, because any symmetric division of LREC would result in the diminishment of ER-positive LRECs, not an increase. Additionally, we previously established that LRECs are in cycle, as demonstrated by the labeling of DNA with a second label (5BrdU) that rapidly disperses to daughter cells [4], proving that LRECs are dividing asymmetrically as they remain labeled with [^3^H]thymidine. Another possibility is that ER and PR initiate different signal transduction pathways and the activation of the ER pathway induces ER production. Potential pathways include the mitogen-activated protein kinase and the phosphatidylinositol-3 kinase pathways [12,13]. A study conducted in the rat indicated that estradiol causes reduction in ER message and protein *in vivo *and *in vitro *in the pituitary [14], which is contrary to our observations. These investigators demonstrated that within the pituitary the different cell types responded differently to estradiol. It is feasible, then, that the murine mammary epithelium responds in a manner different from that of rat pituitary tissue.

Long label retention in cells may result from the presence of originally labeled cells that went out of the cell cycle shortly after label administration. The label-retaining stromal cells shown in Figure 5 represent an example of this circumstance. Label-retaining cells may be represented by cells, which traverse the cell cycle very slowly. Alternatively, in this report, label-retaining cells (LRECs) result from the retention of the originally labeled DNA during asymmetric cell divisions and passage of newly synthesized DNA to the progeny. This distinction may explain the difficulty in producing LRECs in adult mammary tissues because asymmetrically cycling cells would not retain the newly labeled DNA strands. This in part may account for the observation that estradiol-stimulated mammary tissues seldom contain ER-α-positive, [^3^H]thymidine-labeled cells in studies designed to determine the location of cells mitotically responsive to estradiol stimulation.

The present findings indicate that labeled stem cells that formed during allometric growth of the mammary gland express ER or PR. Furthermore ER-α and PR expression in these stem cells is affected by estrogen and progesterone.

## Conclusion

The results presented here support the premise that a subpopulation of LRECs (potentially epithelial stem cells) in the murine mammary gland is ER-α and/or PR, and that expression of these receptors by LRECs may be modulated by exogenous stimuli.

## Abbreviations

5BrdU = 5-bromo-deoxyuridine; ER = estrogen receptor; LREC = label-retaining epithelial cell; PR = progesterone receptor; TGF = transforming growth factor.

## Competing interests

The authors declare that they have no competing interests.

## Authors' contributions

GHS conceived of the study and its design, and interpreted the data. BWB performed collection of the data, statistics and interpretation of the data, and wrote the manuscript. Both authors read and approved the final manuscript.

## Figures and Tables

**Table 1:** Percentages of receptor positive cells in the entire epithelium

Time point	Percentages	
	
	ER-α^+^	PR^+^
Week 6	28.4 ± 2.1	31.8 ± 1.4
Week 11	30.7 ± 1.35	31.6 ± 3.1
Week 11 (group I)	28.3	26.3
Week 11 (group II)	35.0	26.1
Week 11 (group III)	24.2	30.4
Week 12 (group I)	26.4 ± 5.5	25.7 ± 1.6
Week 12 (group II)	24.4 ± 1.7	24.9 ± 2.1
Week 12 (group III)	23.2 ± 2.7	24.9 ± 1.5

One thousand epithelial cells were counted in mammary gland sections stained for ER-α or PR. Group I animals received estradiol injections; group II received estradiol and progesterone; and group III received estradiol, progesterone, and prolactin. ER, estrogen receptor; PR, progesterone receptor.

**Table 2:** Percentages of receptor positive cells within the LREC population

Time point	Percentage	
	
	ER-α^+^	PR^+^
Week 11	27.8	28.5
Group 1 early	45.0	31.1
Group 2 early	23.7	19.1
Group 3 early	29.7	24.7
Group 1 late	22.7	25.9
Group 2 late	26.1	30.8
Group 3 late	31.9	25.5

One thousand epithelial cells were counted in mammary gland sections stained for ER-α or PR. Group I animals received estradiol injections; group II received estradiol and progesterone; and group III received estradiol, progesterone, and prolactin. ER, estrogen receptor; LREC, label-retaining epithelial cell; PR, progesterone receptor.
